# SGLT2 inhibitor dapagliflozin reduces endothelial dysfunction and microvascular damage during cardiac ischemia/reperfusion injury through normalizing the XO-SERCA2-CaMKII-coffilin pathways

**DOI:** 10.7150/thno.75121

**Published:** 2022-06-27

**Authors:** Li Ma, Rongjun Zou, Wanting Shi, Na Zhou, Shaoxian Chen, Hao Zhou, Xinxin Chen, Yueheng Wu

**Affiliations:** 1 Guangdong Provincial Key Laboratory of Research in Structural Birth Defect Disease, Heart Center, Guangzhou Women and Children's Medical Center, Guangzhou Medical University, Guangzhou, China.; 2 Department of Paediatrics, Guangdong Provincial Key Laboratory of Research in Structural Birth Defect Disease, Guangzhou Women and Children's Medical Center, Guangzhou Medical University, Guangzhou 510623, China.; 3 Guangdong Cardiovascular Institute, Guangdong Provincial Key Laboratory of South China Structural Heart Disease, Guangdong Provincial People's Hospital & Guangdong Academy of Medical Sciences, School of Medicine, South China University of Technology, Guangzhou, China.; 4 Senior Department of Cardiology, The Sixth Medical Center of People's Liberation Army General Hospital, Beijing, China.

**Keywords:** Dapagliflozin, microvascular dysfunction, cardiac ischemia/reperfusion injury, XO, SERCA2, CaMKII, cofilin

## Abstract

**Background:** Given the importance of microvascular injury in infarct formation and expansion, development of therapeutic strategies for microvascular protection against myocardial ischemia/reperfusion injury (IRI) is of great interest. Here, we explored the molecular mechanisms underlying the protective effects of the SGLT2 inhibitor dapagliflozin (DAPA) against cardiac microvascular dysfunction mediated by IRI.

**Methods:** DAPA effects were evaluated both *in vivo*, in mice subjected to IRI, and *in vitro*, in human coronary artery endothelial cells (HCAECs) exposed to hypoxia/reoxygenation (H/R). DAPA pretreatment attenuated luminal stenosis, endothelial swelling, and inflammation in cardiac microvessels of IRI-treated mice.

**Results:** In H/R-challenged HCAECs, DAPA treatment improved endothelial barrier function, endothelial nitric oxide synthase (eNOS) activity, and angiogenic capacity, and inhibited H/R-induced apoptosis by preventing cofilin-dependent F-actin depolymerization and cytoskeletal degradation. Inhibition of H/R-induced xanthine oxidase (XO) activation and upregulation, sarco(endo)plasmic reticulum calcium-ATPase 2 (SERCA2) oxidation and inactivation, and cytoplasmic calcium overload was further observed in DAPA-treated HCAECs. DAPA also suppressed calcium/Calmodulin (CaM)-dependent kinase II (CaMKII) activation and cofilin phosphorylation, and preserved cytoskeleton integrity and endothelial cell viability following H/R. Importantly, the beneficial effects of DAPA on cardiac microvascular integrity and endothelial cell survival were largely prevented in IRI-treated SERCA2-knockout mice.

**Conclusions:** These results indicate that DAPA effectively reduces cardiac microvascular damage and endothelial dysfunction during IRI through inhibition of the XO-SERCA2-CaMKII-cofilin pathway.

## Introduction

A growing body of evidence supports a primary role of vascular endothelial cells (ECs) and microvascular dysfunction in the acute cardiomyocyte death that characterizes cardiac ischemia/reperfusion injury (IRI) 1, 2. Microvascular injury compromises the therapeutic effect of revascularization treatments, leading to perfusion defects in 15%-50% of patients receiving percutaneous coronary intervention and coronary artery bypass graft surgery 3. It therefore contributes to infarct formation and expansion, is closely associated with reductions in left ventricular ejection fraction, and negatively impacts prognosis 4, 5. However, current research is mainly focused on molecular mechanisms and preventive strategies targeting cardiomyocyte IRI, whereas insufficient emphasis is placed in the field of microvascular protection.

Dapagliflozin (DAPA) is a sodium-glucose linked transporter 2 (SGLT2) inhibitor used to lower blood sugar levels in patients diagnosed with type-2 diabetes mellitus (T2DM) 6. Unlike insulin or glucagon-like peptide-1, DAPA acts by increasing the excretion of glucose through the urine 7. Results of the DECLARE-TIMI 58 trial indicated that treatment with DAPA is associated with a significant reduction in the risk of major adverse cardiovascular events in patients diagnosed with T2DM 8. Subsequently, the DAPA-HF study reported the protective action of DAPA in patients with established heart failure with reduced ejection fraction, regardless of the presence of T2DM 9. The role of DAPA in improving endothelial dysfunction has also been widely investigated. In a prospective, open-label, single-center, randomized clinical trial, DAPA treatment improved 1-min flow-mediated dilation (FMD) at rest and 1-min FMD after 15 min of ischemia followed by 15 min of reperfusion in patients with T2DM and carotid intima-media thickness above the 75^th^ percentile 10. Experiments in cultured human coronary artery endothelial cells (HCAECs) showed that DAPA neutralized superoxide production and restored NO activity upon stimulation with TNFα 11. In turn, in a rat model of diabetic cardiomyopathy DAPA was shown to ameliorate myocardial fibrosis by preventing endothelial-to-mesenchymal transition 12. Although these findings are indicative of the broad vasculoprotective effects of DAPA, it remains unclear whether DAPA would be effective against IRI-related EC dysfunction and microvascular damage.

Our previous studies have identified oxidative stress injury and abnormal intracellular calcium dynamics as critical mechanisms accounting for IRI-related EC dysfunction and microvascular damage 13-15. Reperfusion-mediated xanthine oxidase (XO) activation generates excessive ROS, which reduces the bioavailability of NO 16, resulting in microvascular contraction. Pathological alterations in intracellular calcium handling include cytoplasmic calcium overload and mitochondrial calcium deposition. Cytoplasmic calcium overload impairs endothelium-dependent vasodilation and induces microvascular spasm, whereas mitochondrial calcium accumulation contributes to ROS generation and opening of the mitochondrial permeability transition pore (mPTP) to activate the caspase-9-dependent apoptosis pathway 17. On the other hand, an important role has been proposed for Ca^2+^/calmodulin-dependent protein kinase II (CaMKII) as a central regulator of multiple cardiac cell death pathways 18, 19. However, the relationship between XO-induced oxidative stress and abnormal intracellular calcium signaling is incompletely understood. Sarco(endo)plasmic reticulum Ca^2+^-ATPase-2 (SERCA2) is the primary mechanism for Ca^2+^ uptake into the SR/ER 20, 21. SERCA2 oxidation at Cys-674 promotes SERCA2 degradation and thus increases baseline intracellular calcium levels 22, 23, a pivotal phenomenon in myocardial dysfunction during heart failure 24, 25. Moreover, decreased SERCA2 expression has been found to contribute to IRI-related microvascular damage 14, 26-28. Based on this evidence, we asked whether IRI promotes XO-mediated SERCA2 oxidation and inactivation, leading to abnormally increased intracellular calcium levels in cardiac microvascular ECs.

A recent study conducted in human coronary artery ECs showed that DAPA reduced stretch-induced endothelial barrier dysfunction by inhibiting the generation of ROS 29. Indeed, several studies in ECs, neurons, and cardiomyocytes confirmed the antioxidant properties of DAPA and focused on its regulatory action on antioxidant enzymes such as GSH and SOD 30, 31. However, it remains unclear whether DAPA can counteract XO-induced oxidative stress in ECs during IRI. Meanwhile, a recent study in diabetic mice with AngII-induced cardiomyopathy showed that DAPA attenuated intracellular calcium transients and downregulated the expression of calcium channels and transporters (i.e. CACNA1C, NCX, and NHE1) in isolated cardiomyocytes 32 However, there is no evidence, to our knowledge, on the potential regulatory influence of DAPA on SERCA2-dependent calcium reuptake in IRI-challenged ECs. In view of the above considerations, the goal of the present study was to explore the molecular mechanisms governing DAPA-mediated cardiac microvascular protection during IRI-induced injury.

## Materials and Methods

### Endothelial-specific SERCA2 knockout mouse and echocardiography

The generation of SERCA2*^f/f^* mice was established based on previous studies 33. Subsequently, Tie2*^Cre^* transgenic mice and SERCA2*^f/f^* mice were extensively backcrossed to C57B6/J mice (Jackson Laboratory, Bar Harbor, ME, USA) for at least nine generations before interbreeding to generate endothelial-specific SERCA2 knockout mice (SERCA2*^EKO^*). SERCA2*^f/f^* mice were used as the control group. To avoid potential gender-related variation, all experiments were performed using male mice.

Mice were anesthetized with 2% Isoflurane and underwent transthoracic echocardiography using the VisualSonics Vevo 2100 Imaging System with a high frequency (30 Mhz) probe. Briefly, mice chests were depilated using Nair crème, and mice were placed supine on a heated board with embedded ECG leads. Temperature was strictly maintained between 36.5 ºC and 37.5 ºC, and heart rate was maintained above 400 beats per minute to void confounding effects of hypothermia and bradycardia. B-mode short axis images and M-mode scans were collected, and analyzed following completion of the data acquisition 34.

### Cardiac ischemia reperfusion injury (IRI) model

Mice were fully anesthetized with isoflurane (1%-1.5%), intubated, and connected to a rodent ventilator. A median sternotomy was performed to access and identify the left anterior descending coronary artery (LAD), which was surgically ligated with a 7-0 silk suture 35. A short segment of PE-10 silicon tubing was placed between the LAD and the described suture to cushion the artery against trauma. Mice were subjected to 45 min of LAD ischemia, followed by reperfusion for 2 h. All mice undergoing LAD occlusion surgery survived until sacrifice (up to 6 h post-surgery). After 2 h of reperfusion, in a subset of mice the heart was rapidly excised for immunohistochemistry analysis. Dapagliflozin (40 mg/kg/day) was administrated once a day via intraperitoneal injection over seven days before IRI surgery.

### Histological analysis

Histological changes in cardiac tissue were evaluated by hematoxylin and eosin (H&E) staining of paraffin-embedded, 5 μm-thick tissue sections 36. ICAM1 (1:12,000 dilution; ab179707, Abcam, Cambridge, UK) immunohistochemistry was performed to assess cardiac inflammation, by quantifying the number positive cells per high-power field. Digital images were acquired with by microscopy (Eclipse 80i, Nikon, Tokyo, Japan).

### Cell culture and H/R model

Human coronary artery endothelial cells (HCAECs; PCS-100-020™, ATCC, Manassas, VA, USA) were cultured in Vascular Cell Basal Medium (ATCC) with 5 ng/ml VEGF, 5 ng/ml EGF, 5 ng/ml FGF, 15 ng/ml IGF-1, 10 mM L-glutamine, 0.75 U/ml heparin sulfate, 1 µg/ml hydrocortisone, 50 µg/ml ascorbic acid, 1% amphotericin B, 1% penicillin-streptomycin, and 10% FBS. Hypoxia-reoxygenation (H/R) injury was induced on cultured cells as described in our previous study 15. In brief, hypoxic stress was induced in a tri-gas incubator with an N_2_ concentration of 95% and a CO_2_ concentration of 5% for 45 min. Then, cells were cultured under normal oxygen concentration for 2 h. Dapagliflozin (10 μM) was applied to HCAECs 24 h before H/R injury *in vitro*. BAPTA (50 μM; #A1076, Sigma-Aldrich, Burlington, MA, USA) was added to the medium for 30 min to attenuate intracellular calcium overload. Ionomycin (100 μM; #200-664-3, Sigma) was alternatively applied for 30 min to induce calcium overload. To inhibit F-actin degradation, HCAECs were treated with jasplakinolide (Jas, 2 μM; #ab141409. Abcam) over 2 h before H/R. To induce an oxidative stress microenvironment, HCAECs were treated with 0.3 mM hydrogen peroxide for 6 h. To prevent CaMKII activation, HCAECs were incubated with KN93 (5 μM; #S678, Selleck Chem. LLC, Houston, TX, USA) during 2 h before H/R.

### shRNA lentiviral vector transduction and siRNA transfection

To silence SERCA2 and XO expression, shRNAs targeting human SERCA2 or XO mRNAs were cloned into the pLKO.1 lentiviral vector (10878, Addgene, Watertown, MA, USA). An empty shRNA hairpin containing GFP was used as control. Target plasmids were co-transfected with packaging plasmids (psPAX2; 12260, Addgene) and pMD2.G (12259, Addgene) into 293T cells. The virus-containing supernatant was harvested 48 h after transfection and filtered through a 0.45 μm filter (SLHV033RB, MilliporeSigma, Burlington, MA, USA) to obtain the corresponding lentiviral particles. These were incorporated into HCAECs using polybrene (H9268; Sigma), and puromycin (A1113803; Gibco, Grand Island, NY, USA) was used for selection of cells with stable SERCA2/XO knockdown (verified by western blotting). The siRNA against SGLT2 (Sense, 5'-GUCAUUGCUGCAUAUUUCCTT-3', Antisense, 5'-GGAAAUAUGCAGCAAUGACTA-3'), were purchased from Thermo Fisher Scientific (#289404, 200 nM, San Jose, CA, USA), and transfected into HCAECs for 48 hrs according to our previous study 37. The knockdown efficiency of siRNA transfection was confirmed by western blots.

### Quantitative real-time PCR

Total RNA was extracted by TRIzol (T9424, Sigma) and purified using the phenol-chloroform method. RNA was reverse transcribed into cDNA using a Transcriptor First-Strand cDNA Synthesis Kit (04896866001, Roche, Basel, Switzerland). SYBR Green (04887352001, Roche) was used in quantitative real-time PCR. PCR amplification conditions were as follows: initialization at 94 °C for 2 minutes; 40 cycles of denaturation at 94 °C for 30 seconds, annealing at 45 °C for 30 seconds, elongation at 72 °C for 105 seconds, and final elongation at 72 °C for 10 minutes. β-actin was used as internal reference gene. Primers sequences were: TNFα (Forward, 5'- AGATGGAGCAACCTAAGGTC-3'; Reverse, 5'-GCAGACCTCGCTGTTCTAGC-3'), IL6 (Forward, 5'-CAGACTCGCGCCTCTAAGGAGT3'; Reverse, 5'-GATAGCCGATCCGTCGAA-3'), MCP1 (Forward, 5'-GGATGGATTGCACAGCCATT-3'; Reverse, 5'-GCGCCGACTCAGAGGTGT-3'), Drp1 (Forward, 5′-TAGTGGGCAGGGACCTTCTT-3′; Reverse, 5′-TGCTTCAACTCCATTTTCTTCTCC-3′); Mff (Forward, 5′-AAGTGGCTCTCACCCTAGCA-3′; Reverse, 5′-TGCCCCACTCACCAAATGT-3′); Mfn2 (Forward, 5′-GCT​CCT​GAA​GGA​TGA​CCT​CG-3′; Reverse, 5′-CGT​CTG​CAT​CAG​CGT​GGA​CTC-3′); Opa1 (Forward, 5′-CAGTGTTGATGACAGCTCAG-3′; Reverse, 5′-CATCACACACTAGCT TACATTTGC -3′).

### Western blotting

Cells were lysed with RIPA buffer (65 mM Tris HCl, 150 mM NaCl, 1 mM EDTA, 1% Nonidet P-40, 0.5% sodium deoxycholate, and 0.1% SDS) with protease inhibitor (04693132001, Roche) and phosphatase inhibitor tablets (4906837001, Roche). Protein concentration was quantified with a BCA kit. Similar amounts of protein were separated by 8-12% SDS-PAGE, transferred to polyvinylidene difluoride (PVDF) membranes (IPVH00010, Millipore), and incubated overnight at 4 °C with corresponding primary antibodies: F-actin (1:1000, Abcam, #ab130935), G-actin (1:1000, Abcam, #ab200046), p-CaMKII (1:1000, Abcam, #ab124880), cofilin (1:1000, Abcam, #ab54532), CaMKII (1:1000, Abcam, #ab52476), p-cofilin (1:1000, Abcam, #ab283500), ET-1 (1:1000, Abcam, #ab178454), eNOS (1:1000, Abcam, #ab199956), p-eNOS (1:1000, Abcam, #ab215717), SGLT2 (:1000, Abcam, #ab37296), Fak (1:1000, Abcam, #ab40794), Src (1:1000, Abcam, #ab133283), GAPDH (1:1000, Abcam, #ab8245), XO (1:1000, Abcam, #ab109235), SERCA2 (1:1000, Abcam, #ab150435), Fak (1:1000, Abcam, #ab40794), Src (1:1000, Abcam, #ab133283), Tom20 (1:1000, Abcam, #ab186735), Drp1 (1:1000, Abcam, #ab184247), and SERCA2 C674-SO_3_H (#A300-BL2103; Bethyl Laboratories, Inc., Montgomery, TX, USA). Suitable secondary antibodies were next applied for 1 h at room temperature. Signals were detected on a ChemiDoc imaging system (Bio-Rad, Hercules, CA, USA).

### Intracellular calcium detection

Intracellular calcium levels were analyzed using Fura-2AM (Molecular Probes) and recorded by confocal microscopy using a 60×/1.42 NA oil immersion objective. Excitation and emission wavelengths of 340/380 nm and 500 nm were respectively set to capture the fluorescence intensity of Fura-2AM in HCAECs. Fura-2AM is advantageous over other Ca^2+^-sensitive indicators because it can be used in the dual excitation ratiometric mode to measure cytoplasmic Ca^2+^ and Fura-2AM has an isofluorescence wavelength (~340 nm) where its fluorescence is independent of Ca^2+^ 38. The ratio of the emissions at 340 nm and 380 nm wavelengths is directly related to the amount of intracellular Ca^2+^ 38. In our study, cells were incubated on the confocal microscopy in the presence of 1 μM Fura-2AM for 2 minutes while monitoring the fluorescence intensities at 340 nm and 380 nm (excitation). The relative fluorescence intensity was normalized to that of the control group 14, 17.

### ATP and reactive oxygen species (ROS) measurements

ATP levels in HCAECs were measured with the ATP Assay Kit (S0026; Beyotime Biotechnology, Haimen, Jiangsu, China) according to the manufacturer's instructions. Total ROS levels in HCAECs were measured using the ROS Detection Cell-Based Assay Kit (601290; Cayman Chem., Ann Harbor, MI, USA) according to the manufacturer's recommendations.

### CCK-8 and MTT assay

Cell proliferation was determined by the CCK-8 Kit (Dojindo Laboratories, Kumamoto, Japan) according to the manufacturer's instructions. Cells were seeded at 3,000 cells per well in 96-well plates. After treatment with DAPA, 10 ml of CCK-8 solution was added to each well, followed by incubation at 37 °C for 1 h. OD values were next measured at 450 nm using a microplate reader. MTT assay was used to determine cell viability as our previously described 14.

### Immunohistochemistry, immunofluorescence, and TUNEL assay

For immunohistochemistry, 4% paraformaldehyde overnight-fixed, paraffin-embedded samples were serially sectioned into 4-μm-thick slices and processed using standard procedures. After autoclave antigen retrieval in citrate buffer, sections were immunostained with an ICAM1 antibody (1:500, Abcam, #ab119871) followed by an HRP-conjugated secondary antibody and then visualized using a 3,**'**3-Diaminobenzidine (DAB) staining kit (RE7230-K; Leica). Nuclei were counterstained with hematoxylin solution. For immunofluorescence, samples were treated with a Gr1 antibody (1:1000, Abcam, #ab25377), followed by incubation with Alexa Fluor 488-conjugated anti-rabbit IgG (1:200, Ab150117), Alexa Fluor 555-conjugated anti-mouse IgG (1:200, Ab150074) antibody. Nuclei were stained with DAPI (Vector). TUNEL staining was performed on cultured HCAECs based on the manufacturer's protocol.

### Enzyme-linked immunosorbent assay and XO inhibitory assay *in vitro*

ELISA quantification kits to analyze the activity of VEGF (Human VEGF ELISA Kit, #ab222510) and XO (Xanthine Oxidase Activity Assay Kit, #ab102422) were purchased from Abcam. In brief, 50 μl of standard solution or samples were added to each well. The plates were incubated for 2 h at RT with continuous shaking. After washing with 200 μl of wash buffer five times, 50 μl of biotinylated albumin antibody was added for 1 h. After washing, 50 μl of streptavidin-peroxidase conjugate was added over 30 min. The plates were then washed and 50 μl of chromogen substrate per well was added for 30 min. When color developed, 50 μl of stop solution was applied. Plates were immediately read at 450 nm on a microplate reader. A standard curve based on serial dilutions of standards was generated to calculate samples' concentrations.

The *in vitro* XO inhibitory assay was performed according to the procedures as previously described 39. In brief, DAPA was added into 0.1 mL of enzyme solution (0.01 units/mL in phosphate buffer, pH 7.5). After pre-incubation at 25 °C for 15 min, the reaction was initiated by the addition of 2 mL of 150 mM xanthine solution in the same buffer which acts as a substrate and this reaction mixture were incubated at 25 °C for 30 min 40. Then, 1 mL of 1 nM HCL wad added into the solution to stop the reaction and the absorbance was measured at 290 nm by using a UV spectrophotometer. One unit of XO is defined as the mount of enzyme required to produce 1 mmol of uric acid per min at 25 °C 40.

### SERCA2 activity assay

SERCA2 activity was measured using the colorimetric ATPase assay kit (catalogue no. 601-0120, Novus Biologicals, Littleton, CO) as our previously described 41. In brief, DAPA-treated HCAECs were digested, centrifuged, and then disrupted by an ultrasonic pulverizer. Cellular homogenates (50 μg) were firstly incubated with the ionophore A23187 (1 μg/ml) and EGTA (1μg/ml) for 5 min to prevent a buildup of Ca^2+^ inside the vesicles that might inhibit the Ca^2+^-ATPase activity. The activity rates were read at 650 nm using a microplate reader (Epoch 2; BioTek Instruments, Inc.) and normalized to total protein content measured by MicroBCA protein assay (Pierce).

### Statistical analysis

Data are presented as mean ± SEM. The distributions of all continuous variables were tested for normality assumptions using the D**'**Agostino & Pearson normality test in GraphPad Prism software. One-way analysis of variance (ANOVA) evaluated differences for parameters measured at a single time point within the sham or DAPA group using Dunnett**'**s multiple comparison test. One-way ANOVA with Tukey**'**s multiple comparison test was used for *in vitro* data. Statistical analyses were performed using SAS 9.4 (SAS Institute, Cary, NC) and GraphPad Prism version 7.0. A p < 0.05 was used to determine significance for all statistical tests.

## Results

### DAPA attenuates IRI-induced microvascular injury

**Figure 1:**
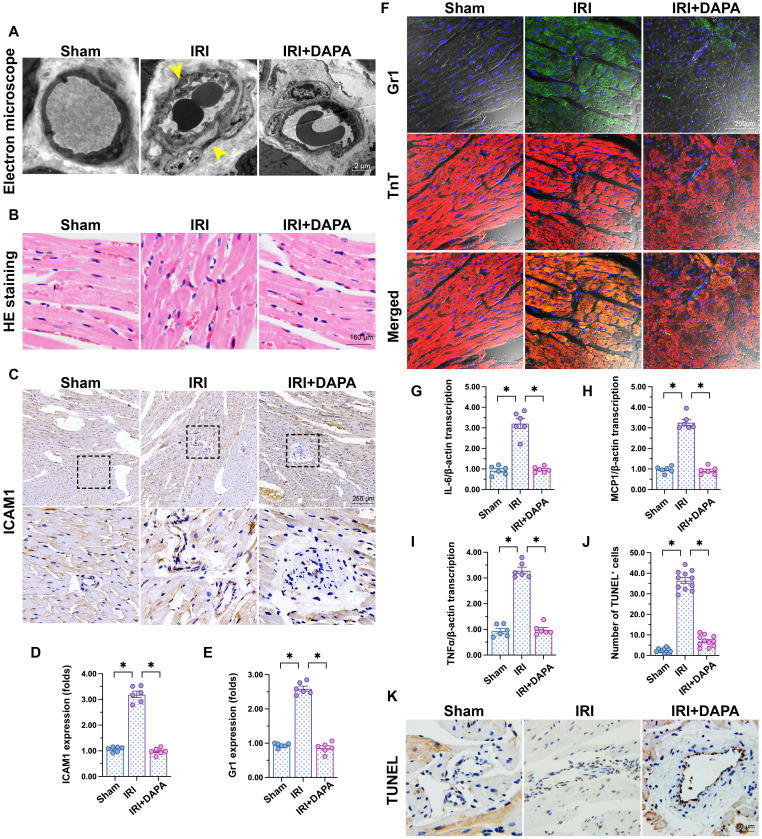
** DAPA attenuates IRI-induced microvascular injury.** Mice were subjected to 45-min ischemia followed by 2-h reperfusion to induce cardiac ischemia/reperfusion injury (IRI). Dapagliflozin (DAPA, 40 mg/kg/day) was administrated daily via intraperitoneal injection during seven days before IRI surgery. **(A)** Electron microscopy was used to detect ultrastructural alterations in the cardiac microcirculation. Yellow arrows indicate narrowed lumens and rough microvessel walls. **(B)** H&E staining was used to observe the morphology of erythrocytes in the cardiac microvasculature.** (C, D)** Immunohistochemistry was performed on heart tissues to detect the expression of ICAM1 on the surface of cardiac microvessels. **(E, F)** Immunofluorescence was used to detect intracardiac accumulation of Gr1^+^ neutrophils. Cardiomyocytes were stained with TnT and nuclei were counterstained with DAPI. **(G-I)** The expression of IL-6, MCP1, and TNFα mRNA was determined by qPCR. β-actin was used as internal reference. **(J, K)** TUNEL staining was performed to detect and quantify apoptosis of cardiac microvascular ECs after IRI. Experiments were repeated at least three times and the data are shown as mean ± SEM. Six animals were used in each group and the dotes in each panel represent the average data of three replicates in each animal. *p < 0.05.

To investigate the influence of DAPA on IRI-related microvascular injury, we first applied electron microscopy to observe cardiac microvascular ultrastructural changes in mice subjected to 45-min ischemia followed by 2 h of reperfusion to induce cardiac IRI. As shown in Figure 1A, swollen ECs, rough microvessel walls, and narrowed lumens were observed in the IRI control group. However, DAPA protected against IRI-induced structural changes. In samples from IRI-treated control mice, erythrocytes adopted a pie-like shape morphology consistent with luminal stenosis and hemodynamic disorder (Figure 1B). Notably, normal erythrocyte features were largely preserved after DAPA pretreatment (Figure 1B). Furthermore, immunohistochemistry analysis demonstrated that IRI promoted the expression of ICAM1, an adhesion molecule that facilitates recruitment of circulating inflammatory cells, in cardiac tissue (Figure 1C-D). Paralleling increased ICAM1 expression, in response to IRI injury numerous Gr1^+^ neutrophils were detected in the myocardium of control animals (Figure 1E-F). Consistent with these findings, the expression of pro-inflammatory factors (IL-6, MCP1, and TNFα) was also upregulated in response to IRI injury (Figure 1G-I). Treatment with DAPA not only reduced ICAM1 expression but also prevented the infiltration of circulatory neutrophils into heart tissues and repressed the transcription of the above cytokines (Figure 1E-I). Endothelial damage was further assessed through TUNEL staining. IRI increased the number of TUNEL positive ECs and this effect was prevented by DAPA (Figure 1J-K). Lastly, echocardiography demonstrated that cardiac systolic/diastolic function was impaired by IRI and reversed to near-normal levels with DAPA treatment (Table 1). These findings indicate that DAPA prevents morphological alterations and counteracts inflammation caused by IRI in the cardiac microcirculation.

### DAPA sustains cardiac microvascular endothelial function during IRI

**Figure 2:**
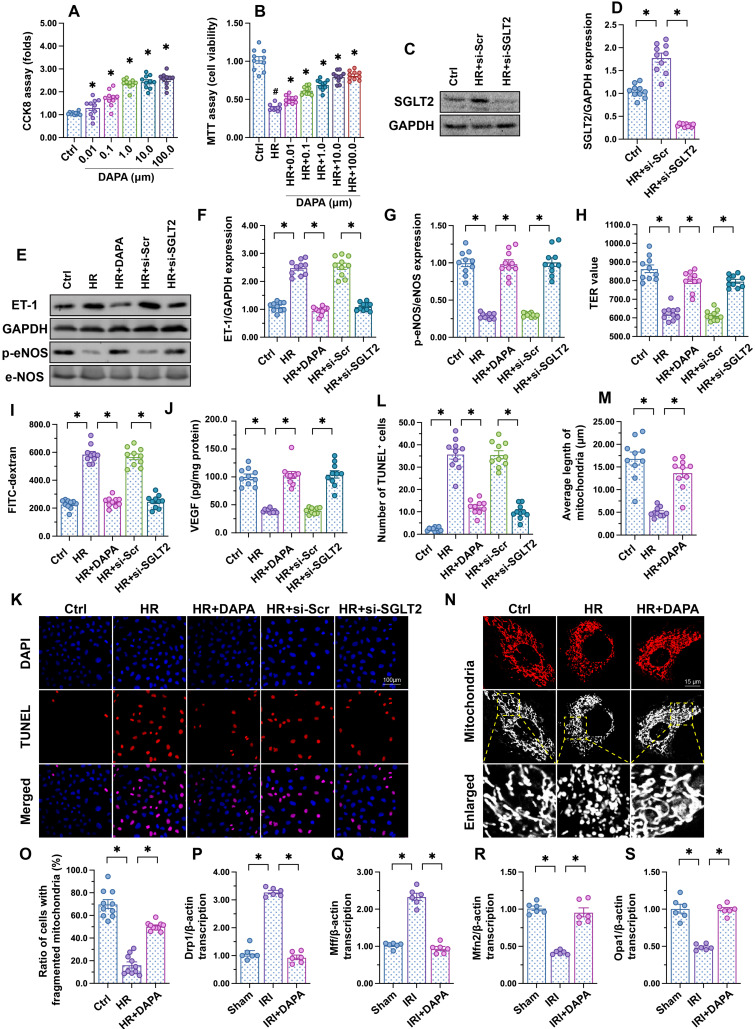
** DAPA sustains endothelial function during IRI.** Human coronary artery endothelial cells (HCAECs) were treated with 45-min hypoxia (H) followed by a 2-h reoxygenation (R) phase to induce H/R injury. **(A)** HCAECs were treated with different doses of DAPA and cell proliferation was analyzed by CCK-8 assay. *p < 0.05 vs. control group. **(B)** HCAECs were treated with different concentrations of DAPA in the presence of H/R injury. Cell viability was determined by MTT assay. #p < 0.05 vs. control group, *p < 0.05 vs. H/R group. **(C-D)** Western blots were used to observe the expression of SGLT2 in HCAECs. siRNA against SGLT2 (si-SGLT2) and the scrambled siRNA (si-Scr) were transfected into HCAECs to knockdown the expression of SGLT2. **(E-G)** Western blot analysis of alterations in ET-1 and p-eNOS expression. DAPA (10 µM) was applied to HCAECs 24 h before H/R*.* siRNA against SGLT2 (si-SGLT2) and the scrambled siRNA (si-Scr) were transfected into HCAECs to knockdown the expression of SGLT2. **(H, I)** Endothelial barrier function and permeability were determined by fluorescein isothiocyanate (FITC)-dextran clearance and transendothelial electrical resistance (TER) assays. DAPA (10 µM) was applied to HCAECs 24 h before H/R*.* siRNA against SGLT2 (si-SGLT2) and the scrambled siRNA (si-Scr) were transfected into HCAECs to knockdown the expression of SGLT2. **(J)** ELISA was used to quantify VEGF release by HCAECs. **(K-L)** Cell apoptosis was determined by TUNEL staining, respectively. **(M-O)** Immunofluorescence assay of mitochondrial morphology using the TOM20 antibody. The average length of mitochondria and the ratio of cells with fragmented mitochondria was recorded. **(P-S)** Western blots analysis of mitochondrial fission/fusion-related proteins in heart tissues. Experiments were repeated at least three times and the data are shown as mean ± SEM (n = ten independent cell isolations per group). *p < 0.05.

To investigate the molecular basis of DAPA-mediated protection against IRI-induced microvascular dysfunction, cultured human cardiac artery endothelial cells (HCAECs) were treated with different concentrations of DAPA to evaluate its toxic effects* in vitro*. The CCK8 assay confirmed that DAPA dose-dependently promoted HCAECs proliferation (Figure 2A). After exposure to hypoxia-reoxygenation (H/R), HCAECs viability, as measured by MTT assay, was significantly reduced (Figure 2B). However, DAPA maintained cell viability in a dose-dependently manner (Figure 2B). These data confirmed that DAPA may protect endothelial cells viability against H/R injury. Since DAPA is an inhibitor of SGLT2, we asked whether DAPA-mediated endothelial protection is achieved via inhibiting the SGLT2 pathway. Western blots confirmed the existence of SGLT2 in HCAECs while H/R treatment slightly elevated the expression of SGLT2 in endothelial cells (Figure 2C-D). The siRNA against SGLT2 (si-SGLT2) was transfected into HCAECs to simulate the inhibitory effect offered by DAPA on SGLT2 pathway (Figure 2C-D). Subsequently, endothelial function was determined in response to DAPA treatment or SGLT2 knockdown. Western blot analysis demonstrated that upon H/R stress eNOS activity was reduced, whereas the expression of ET-1 was upregulated, and these alterations were attenuated by DAPA or si-SGLT2 (Figure 2E-G). To observe changes in endothelial barrier function, fluorescein isothiocyanate (FITC)-dextran clearance and transendothelial electrical resistance (TER) assays were conducted. Cellular hyperpermeability, evidenced by enhanced FITC-dextran deposition and reduced TER, was observed after H/R exposure, and these effects were effectively attenuated by DAPA pretreatment or si-SGLT2 (Figure 2H-I). In addition, decreased VEGF levels were detected in the supernatant of H/R-exposed HCAECs by ELISA (Figure 2J). Notably, VEGF expression could be restored by DAPA or si-SGLT2 (Figure 2J). Lastly, cell apoptosis was determined in HCAECs by TUNEL assays. Results showed that DAPA or si-SGLT2 effectively counteracted the extent of apoptosis elicited by H/R (Figure 2K-L).

Recent studies have reported that mitochondrial fission or fusion may be a potential target of DAPA 42, 43. The inhibitory effect of DAPA on mitochondrial fission contributes to increased endothelial survival and improved coronary function in diabetic mice 44. Therefore, we asked whether DAPA may attenuate H/R-mediated endothelial damage through restoring the balance between mitochondrial fission and fusion. *In vitro*, immunofluorescence staining of mitochondrial morphology illustrated that H/R induced the formation of fragmented mitochondria (Figure 2M-O), an effect that was followed by decreased average length of mitochondria (Figure 2M-O). Treatment with DAPA or si-SGLT2 significantly reversed the mitochondrial morphology in the presence of H/R injury. At the molecular levels, qPCR assay further showed that the transcription of Drp1 and Mff, the regulators of mitochondrial fission, were significantly elevated in IRI-treated heart tissues (Figure 2P-S). By comparison, mitochondrial fusion factors, such as Opa1 and Mfn2, were markedly downregulated in IRI-treated mice (Figure 2P-S). Pretreatment with DAPA inhibited the expression of mitochondrial fission factors and reversed the levels of mitochondrial fusion regulators (Figure 2P-S). Taken together, these results illustrated that mitochondrial morphology, especially mitochondrial fission and fusion, could be normalized by DAPA in the setting of cardiac microvascular reperfusion injury.

### DAPA attenuates endothelial apoptosis by regulating the CaMKII/cofilin pathway

**Figure 3:**
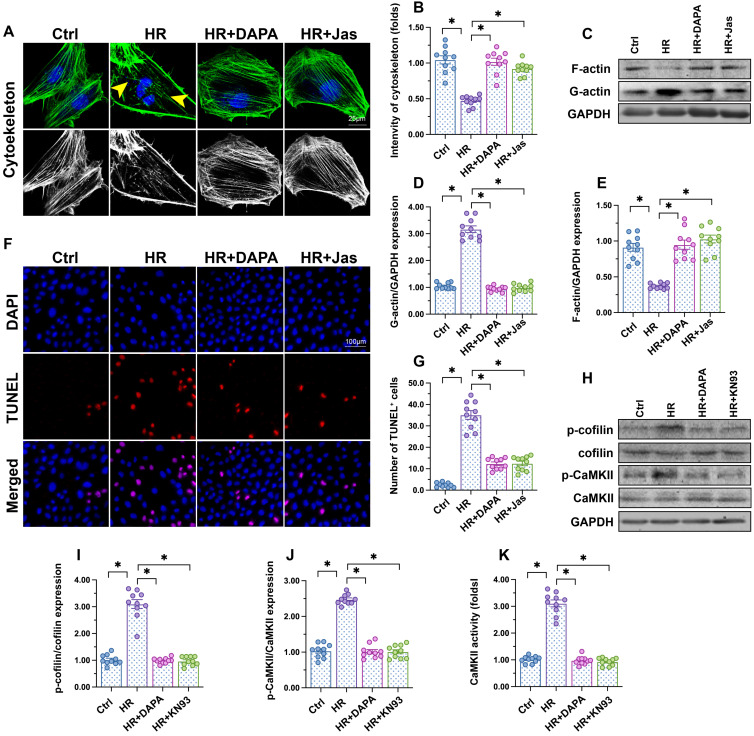
** DAPA attenuates endothelial apoptosis by inhibiting the CaMKII/cofilin pathway.** Human coronary artery endothelial cells (HCAECs) were treated with 45-min hypoxia (H) followed by a 2-h reoxygenation (R) phase to induce H/R injury. DAPA (10 µM) was applied to HCAECs 24 h before H/R*.* **(A, B)** F-actin immunofluorescence was used to observe cytoskeletal changes in HCAECs exposed to H/R injury. Yellow arrows indicate cytoskeleton degradation. Jasplakinolide (Jas, 2 µM), a depolymerizing agent targeting F-actin, was applied to HCAECs 2 h before H/R exposure. **(C-E)** Detection of F-actin and G-actin expression by western blotting. **(F, G)** Apoptosis in HCAECs was assessed through TUNEL staining. **(H-J)** Analysis of alterations in p-cofilin and p-CaMKII expression by western blotting. KN93 (5 µM), an inhibitor of CaMKII, was applied to HCAECs 2 h before H/R treatment.** (K)** ELISA was used to determine CaMKII activity. Experiments were repeated at least three times and the data are shown as mean ± SEM (n = ten independent cell isolations per group). *p < 0.05.

Cytoskeletal degradation is considered an early hallmark of apoptosis induction 45. F-actin immunofluorescence in HCAECs showed a disorganized cytoskeleton after exposure to H/R (Figure 3A-B). Moreover, western blots demonstrated that polymeric F-actin was degraded into monomeric G-actin upon H/R injury (Figure 3C-E). In turn, administration of DAPA sustained cytoskeletal integrity (Figure 3A, 3B) and preserved F-actin expression (Figure 3C-E). Mimicking the results observed with DAPA, application of jasplakinolide (Jas), an actin-stabilizing agent, inhibited cytoskeletal degradation (Figure 3A-B), prevented F-actin disassembling (Figure 3C-E), and reduced the number of TUNEL-positive cells (Figure 3F-G) following H/R. These results suggested that the antiapoptotic effect of DAPA on H/R-challenged vascular endothelial cells is associated with preservation of cytoskeletal homeostasis.

F-actin depolymerization and cytoskeletal degradation are closely associated with cofilin activity 46. Recently studies by our group 47 and other researchers 48 showed that assembling of G-actin into F-actin polymers is sustained by cofilin dephosphorylation. Our western blot results indicated that H/R injury promoted cofilin phosphorylation, a conformational alteration that was attenuated by DAPA (Figure 3H-J). To investigate the mechanism by which DAPA prevents H/R-mediated cofilin phosphorylation, we focused on CaMKII, which has been reported to induce cofilin phosphorylation at Ser-3 49. After exposure to H/R injury, CaMKII activity was significantly increased, as evidenced by upregulated levels of p-CaMKII (Figure 3H-J). Treatment with either DAPA or KN-93 (an inhibitor of CaMKII) was able to prevent H/R-mediated CaMKII phosphorylation, an effect paralleled by decreased cofilin phosphorylation (Figure 3H-J). Furthermore, ELISA demonstrated that the activity of CaMKII was increased by H/R and inhibited by DAPA treatment (Figure 3K). These results indicate that DAPA inhibits CaMKII-mediated cofilin phosphorylation and thus sustains F-actin homeostasis and cytoskeleton integrity in cardiac microvascular endothelial cells subjected to H/R injury.

### DAPA inhibits XO-mediated SERCA2 oxidation and normalizes intracellular calcium balance

**Figure 4:**
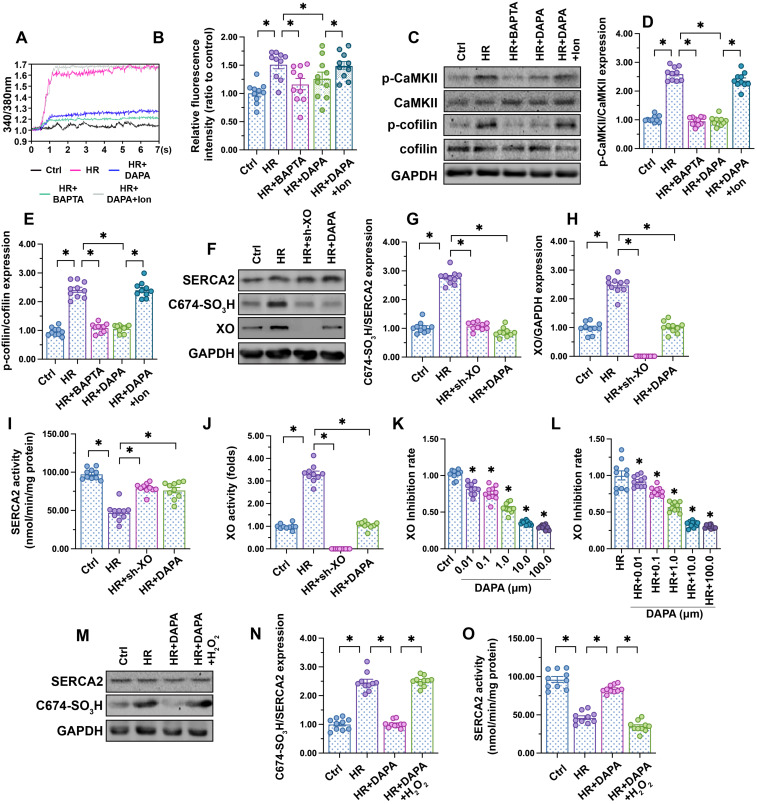
** DAPA inhibits XO-mediated SERCA2 oxidation and normalizes intracellular calcium balance.** Human coronary artery endothelial cells (HCAECs) were treated with 45-min hypoxia (H) followed by a 2-h reoxygenation (R) phase to induce H/R injury. DAPA (10 µM) was applied to HCAECs 24 h before H/R*.*** (A, B)** HCAECs were incubated on the confocal microscopy in the presence of 1 µM Fura-2AM for 2 minutes while monitoring the fluorescence intensities at 340 nm and 380 nm (excitation). The ratio of the emissions at 340 nm and 380 nm wavelengths is directly related to the amount of intracellular Ca^2+^. The relative fluorescence intensity was normalized to that of the control group. HCAECs were treated with BAPTA (50 µM) or ionomycin (Ion, 100 µM) for 30 min before H/R to prevent and induce calcium overload, respectively. **(C-E)** Western blot analysis of p-cofilin and p-CaMKII expression in HCAECs. **(F-H)** Western blot analysis of SERCA2 oxidation (Cys-674) and XO expression. Lentiviral shRNA targeting XO was transduced into HCAECs and knockout efficiency confirmed by western blotting. **(I, J)** ELISA was used to analyze changes in SERCA2 and XO activities. **(K-L)** XO inhibitory activity via *in vitro* system. *p < 0.05 vs. control group or H/R group. **(M-N)** Western blot analysis of SERCA2 oxidation (Cys-674). To induce a pro-oxidative microenvironment, HCAECs were treated with 0.3 mM hydrogen peroxide for 6 h before DAPA treatment. **(O)** ELISA was used to analyze changes in SERCA2 activity. To induce a pro-oxidative microenvironment, HCAECs were treated with 0.3 mM hydrogen peroxide for 6 h before DAPA treatment. Experiments were repeated at least three times and the data are shown as mean ± SEM (n = ten independent cell isolations per group). *p < 0.05.

Fluctuations in intracellular calcium signaling regulate CaMKII activation 50. Experiments in HCAECs using the calcium-sensitive probe Fura-2AM showed an elevation in cytoplasmic calcium levels following H/R injury, and attenuation of this response in DAPA-treated cells (Figure 4A-B). To confirm that calcium overload mediates CaMKII activation, H/R-induced intracellular calcium fluctuations and CaMKII/cofilin phosphorylation were analyzed in HCAECs following addition of BAPTA (a calcium chelator). In separate experiments, the effect of ionomycin (Ion, a calcium agonist), was evaluated in H/R-challenged cells pretreated with DAPA. Results showed that H/R-mediated calcium overload could be attenuated by BAPTA (Figure 4A-B), an effect paralleled by a drop in CaMKII/cofilin phosphorylation (Figure 4C-E). As expected, the attenuating effects of DAPA on H/R-induced calcium elevation and CaMKII/cofilin phosphorylation were nullified by Ion (Figure 4A-E). These findings indicate that DAPA promotes CaMKII inactivation through repressing calcium overload in H/R-exposed arterial endothelial cells.

SERCA2 is the only known molecular pump mediating calcium reuptake and is thus critically responsible for buffering intracellular calcium transients 51. Oxidation of SERCA2 promotes its inactivation, a phenomenon that contributes to cardiomyocyte stiffness during heart failure 24, 25. Western blotting (Figure 4F-H) showed that H/R promoted SERCA2 oxidation at Cys-674, an effect that was accompanied with a drop in SERCA activity (Figure 4I). However, DAPA treatment prevented H/R-mediated SERCA2 oxidation (Figure 4F-H) and thus sustained its activity (Figure 4I). To assess the upstream mechanism involved in the regulation of SERCA2 oxidation, we focused on XO, an enzyme that according to our previous studies is involved in oxidative stress and calcium overload in ECs 14, 16, 41. Western blot analysis demonstrated that XO expression was rapidly upregulated after exposure to H/R injury (Figure 4F-H), an alteration that was followed by an increase in XO activity (Figure 4J). DAPA administration not only prevented XO upregulation (Figure 4F-H) but also inhibited its activity (Figure 4J) in HACEs in the presence of H/R injury. The *in vitro* XO inhibitory assay demonstrated that DAPA dose-dependently reduced XO activity not only under physiological condition (Figure 4K) but also upon H/R exposure (Figure 4I).

To understand whether XO inhibition accounts for DAPA-mediated endothelial protection, sh-RNA against XO was used. Knockout of XO was able to prevent H/R-mediated SERCA2 oxidation (Figure 4F-H) and activation (Figure 4I) in cultured HCAECs. In turn, exogenous supplementation of hydrogen peroxide, to mimic oxidative stress, abolished the inhibitory effect of DAPA on SERCA2 oxidation (Figure 4M-N) and suppressed SERCA2 activity (Figure 4O). These data suggest that DAPA restricts H/R-induced calcium overload in ECs by preventing XO-induced SERCA2 oxidation and inactivation.

### DAPA-mediated endothelial protection is abrogated by SERCA2 knockdown

**Figure 5:**
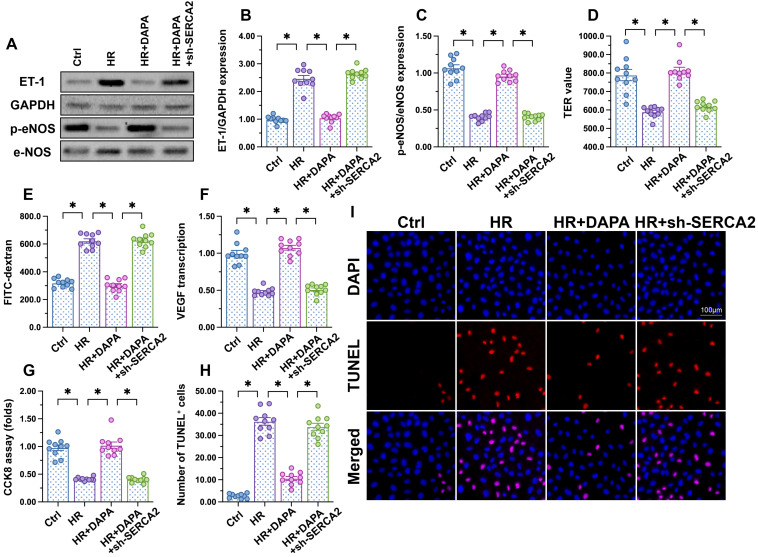
** DAPA-mediated endothelial protection is abrogated by SERCA2 knockdown.** Human coronary artery endothelial cells (HCAECs) were treated with 45-min hypoxia (H) followed by a 2-h reoxygenation (R) phase to induce H/R injury. DAPA (10 µM) was applied to HCAECs 24 h before H/R*.* Lentiviral shRNA targeting SERCA2 was transduced into HCAECs prior to H/R. **(A-C)** Western blot analysis of ET-1 and p-eNOS expression.** (D, E)** Endothelial barrier function and permeability were determined by fluorescein isothiocyanate (FITC)-dextran clearance and TER assays. **(F)** ELISA was used to quantify VEGF release by HCAECs. **(G-I)** Cell viability and apoptosis were determined by CCK8 assay and TUNEL staining, respectively. Experiments were repeated at least three times and the data are shown as mean ± SEM (n = ten independent cell isolations per group). *p < 0.05.

To further assess whether DAPA-mediated protection against H/R injury in ECs is mediated by SERCA2-mediated calcium buffering, loss-of-function assays were performed in H/R-challenged, DAPA-treated HCAECs. Western blot analysis showed that DAPA treatment inhibited ET-1 expression and preserved eNOS activity, and these effects were abrogated upon transduction of lentiviral shRNA vectors targeting SERCA2 (Figure 5A-5C). Moreover, SERCA2 silencing abolished DAPA-mediated normalization of endothelial barrier function and integrity in H/R-exposed cells (Figure 5D, 5E). We further observed that SERCA2 silencing completely inhibited the rescuing effect of DAPA on VEGF synthesis (Figure 5F), and abrogated also its antiapoptotic effect on H/R-treated HCAECs (Figure 5G-5I). These results confirmed that DAPA attenuates H/R-induced microvascular endothelial damage by preventing SERCA2 inactivation.

### DAPA-mediated microvascular protection is compromised in endothelial-specific SERCA2 knockout mice

**Figure 6:**
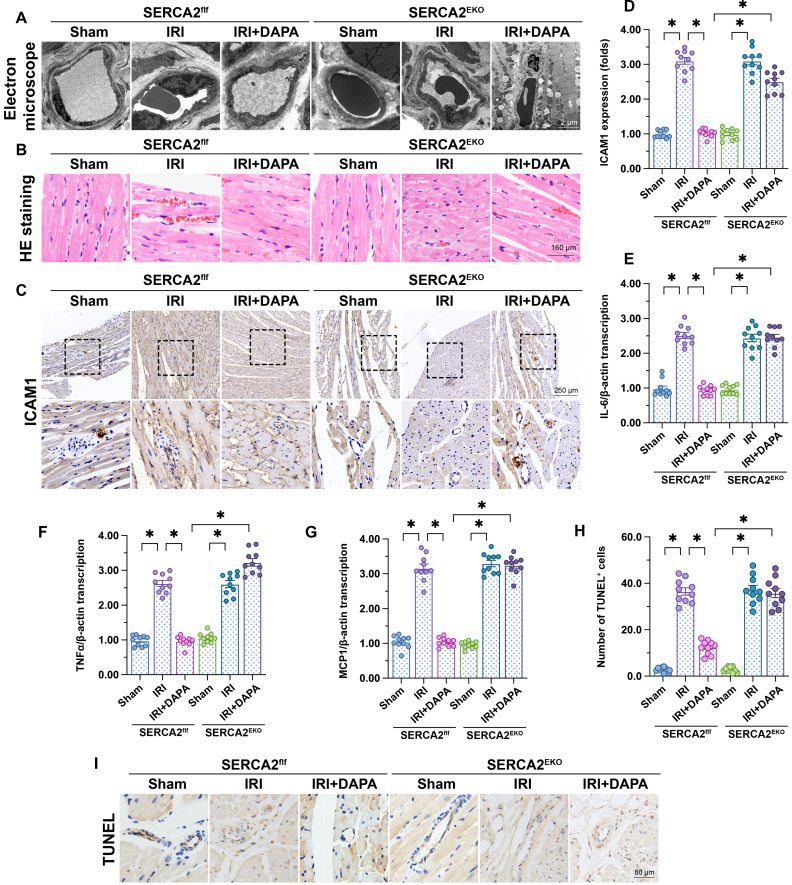
** DAPA-mediated microvascular protection is partly compromised in SERCA2*^EKO^* mice.** Tie2*^Cre^* transgenic mice and SERCA2*^f/f^* mice were extensively backcrossed on C57B6/J mice for at least nine generations before interbreeding to generate the endothelial specific SERCA2 knockout mice (SERCA2*^EKO^*). The SERCA2*^f/f^* mice were used as the control group. Mice were subjected to 45-min ischemia followed by 2-h reperfusion to induce cardiac ischemia/reperfusion injury (IRI). Dapagliflozin (DAPA, 40 mg/kg/day) was administrated daily via intraperitoneal injection during seven days before IRI surgery.** (A)** Electron microscopy was used to detect ultrastructural alterations in the cardiac microcirculation. **(B)** H&E staining was used to observe the morphology of erythrocytes in the cardiac microvasculature.** (C, D)** Immunohistochemistry was performed to detect the expression of ICAM1 on the surface of cardiac microvessels. **(E-G)** The expression of IL-6, MCP1, and TNFα mRNA was determined by qPCR. β-actin was used as internal reference. **(H-I)** TUNEL staining was performed to assess and quantify apoptosis of cardiac microvascular ECs after IRI. Experiments were repeated at least three times and the data are shown as mean ± SEM. Ten animals were used in each group and the dotes in each panel represent the average data of three replicates in each animal. *p < 0.05.

To verify our *in vitro* findings, myocardial IRI was induced in endothelial-specific SERCA2 knockout (SERCA2*^EKO^*) mice. The SERCA2*^f/f^* mice were used as the control group. Electron microscopy showed that knockout of the SERCA2 gene in the vascular endothelium did not affect normal microvascular structure in control mice (Figure 6A). However, DAPA pretreatment failed to improve IRI-induced EC swelling and luminal stenosis in SERCA2*^EKO^* mice (Figure 6A). Furthermore, following IRI, DAPA sustained normal erythrocyte morphology in SERCA2*^f/f^* mice, but not in SERCA2*^EKO^* mice (Figure 6B). In turn, immunohistochemistry analysis of surface ICAM1 expression in cardiac microvessels (Figure 6C, 6D), and qPCR assays assessing the transcription of pro-inflammation factors (Figure 6E-6G) revealed that the anti-inflammatory effect of DAPA was indiscernible in SERCA2*^EKO^* mice. Confirming the crucial involvement of SERCA2 in DAPA-mediated cardiovascular protection against IRI, TUNEL staining showed that the antiapoptotic effect of DAPA was unnoticeable in IRI-exposed SERCA2*^EKO^* mice (Figure 6H, 6I).

## Discussion

**Figure 7:**
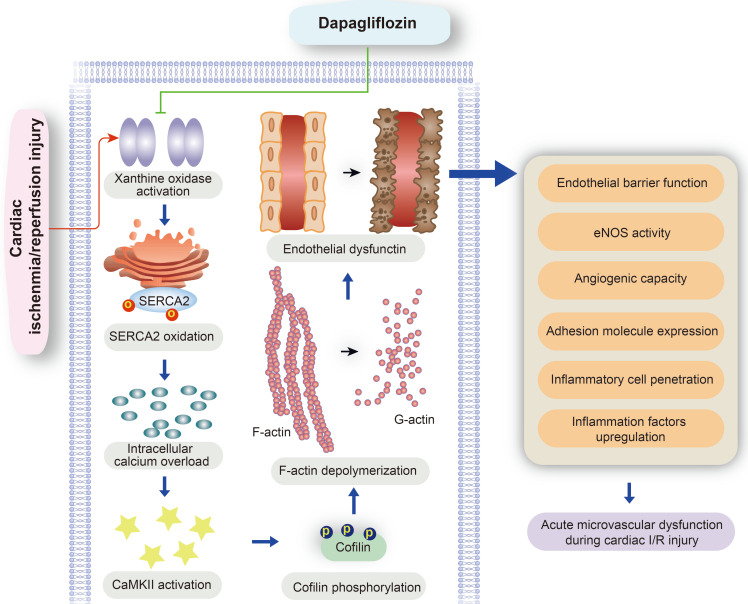
** Protective mechanism of DAPA on cardiac microvascular ischemia reperfusion injury.** DAPA reduced XO expression and activity and inhibited reperfusion-mediated SERCA2 oxidation and inactivation. SERCA2 stabilization inhibited intracellular calcium overload and thus prevented CaMKII-mediated cofilin phosphorylation, resulting in preserved cytoskeleton integrity and decreased endothelial apoptosis. These beneficial effects of DAPA contribute to improved microvascular integrity in the presence of cardiac ischemia reperfusion (I/R) injury.

Our study explored the protective effects of DAPA against microvascular injury and endothelial dysfunction caused by myocardial IRI. The main findings showed that DAPA attenuates IRI-induced endothelial swelling, luminal stenosis, and morphological alterations in erythrocytes; preserves endothelial barrier function and endothelial permeability, preventing activation of the inflammatory response and inflammatory cell infiltration; and sustains cardiac microvascular EC survival, preserves eNOS activity, and promotes angiogenesis. Furthermore, molecular assays showed that DAPA inhibits H/R-induced XO activation and upregulation, which reduces SERCA2 oxidation and inactivation and prevents cytoplasmic calcium overload. By normalizing intracellular calcium balance, DAPA also suppresses CaMKII activation and cofilin phosphorylation, contributing to cytoskeleton integrity and endothelial survival. Thus, our findings indicate that the XO-SERCA2-CaMKII-cofilin pathway is a potential therapeutic target to preserve endothelial viability in the setting of IRI, and suggest that DAPA administration may counteract reperfusion-related microvascular damage through attenuation of oxidative stress, normalization of intracellular calcium homeostasis, and inhibition of apoptosis (Figure 7).

Several studies have reported the beneficial effects of SGLT2 inhibitors on endothelial function. In LPS-treated HCAECs, canagliflozin inhibited IL-6 release, reduced the expression of the glycolytic enzyme hexokinase II (HKII), and increased endothelial viability 52. Indicative of the antioxidant properties of DAPA during exertion of mechanical tension on vascular ECs, a study reported that DAPA alleviates cyclic stretch-induced endothelial permeability in HCAECs through reducing NADPH oxidase-mediated ROS production 29. Meanwhile, a study conducted in a mouse model of diabetes-associated hindlimb ischemia showed that DAPA administration promoted vascular EC proliferation and migration, as well as secretion of multiple angiogenic factors, resulting into increased neovascularization and blood perfusion 53. These results support the potential of SGLT2 inhibitors such as DAPA for therapeutic angiogenesis. Although the underlying molecular mechanisms were not identified, a study in human aortic endothelial cells indicated that cellular senescence and disfunction induced by the tyrosine kinase inhibitor ponatinib could also be rescued by DAPA 54. More importantly, accumulating evidence from clinical studies in diabetic patients further support the therapeutic efficacy of DAPA in maintaining endothelial cell homeostasis and attenuating microvascular injury 55-57. In accordance with these findings, the present results revealed multiple beneficial (antioxidative, anti-inflammatory, and antiapoptotic) effects afforded by DAPA on cardiac microvascular ECs exposed to IRI, and provides a detailed account of the molecular mechanisms involved.

Our cellular and molecular assays showed that DAPA exerts antioxidant actions and prevents H/R-induced intracellular calcium overload by inhibiting XO-mediated oxidation of SERCA2. Since SGLT2 is an electrogenic Na^+^/glucose co-transporter, inhibition of SGLT2 thus might have a hyperpolarizing effect which could lead to less activation of voltage sensitive Ca^2+^-channels and hence lower cytosolic Ca^2+^ levels 58. This could be an additional contributing effect of DAPA on the homeostasis of cytosolic calcium. The regulatory action of DAPA on calcium inhibits CaMKII-mediated cofilin phosphorylation, actin depolymerization, and endothelial apoptosis. Increased EC survival reduces in turn microvascular permeability and attenuates myocardial inflammation in response to H/R. Of note, it recently has been shown that SGLT2i restores coronary flow velocity reserve in a rodent model for obesity and heart failure possibly via effects on endothelium and NO-pathway 59. These actions highlight the potential therapeutic application of DAPA to modulate multiple aspects, such as oxidative stress, calcium imbalance, apoptosis, and inflammation, of ECs' pathological responses to heart failure.

Consistent with previous reports 28, 60, our recent studies highlighted the necessary role played by SERCA2 in improving mitochondrial quality control in microvascular IRI 13, 14. In the present study, we found that DAPA-mediated protection was offset by SERCA2 deletion both *in vivo* and *in vitro*, thus identifying SERCA2 as a novel downstream target of DAPA. SERCA2 downregulation, resulting from its oxidation, induces intracellular calcium overload and impairs EC migration 61. Indeed, endothelial barrier function 62, cGMP-dependent endothelial relaxation 63, and angiogenesis 27 could all be improved through blockade of SERCA2 oxidation. Considering the critical role played by SERCA2 in regulating multiple pathophysiological parameters in ECs, our findings suggest that DAPA might be of great clinical value to reduce the oxidative status of SERCA2. In this regard, and in light of the indispensable role played by SERCA2 oxidation/inactivation in heart failure 24, 25, 64, it would be of interest to evaluate whether SERCA2 stability may account for the cardioprotection exhibited by DAPA in patients with heart failure 10.

Besides, we also reported the protective effects offered by DAPA on mitochondrial morphology. Mitochondrial dysfunction has been regarded as a potential mechanism involving the progression of cardiac microvascular reperfusion injury 15, 35, 65. Abnormal mitochondrial fission, decreased mitophagy, and excessive mitochondrial oxidative stress contribute to the endothelial dysfunction or death during cardiac reperfusion attack 15, 35, 65. In the present study, we found that DAPA treatment was associated a drop in mitochondrial fission, an effect that was followed by normalized mitochondrial network. At the molecular levels, DAPA administration prevented reperfusion-induced Drp1/Mff upregulation and reversed the levels of Opa1/Mfn2, leading into decreased formation of fragmented mitochondria. The regulatory mechanism of DAPA on mitochondrial fission/fusion is likely associated with Sirt1 or AMPK based on recent in-depth studies 66, 67. It has been also found that DAPA reduced reperfusion-caused myocardial injury through improving mitochondrial function, biogenesis and dynamics 68, suggesting that mitochondria are the downstream target of DAPA. Therefore, the beneficial actions of DAPA on mitochondria may also reduce the vulnerability of endothelial cells to cardiac reperfusion injury.

In summary, this is to our knowledge the first study to describe the beneficial role and mechanism of action of DAPA against microvascular injury and endothelial dysfunction induced by myocardial IRI. Mechanistically, DAPA reduced XO expression and activity and inhibited reperfusion-mediated SERCA2 oxidation and inactivation. SERCA2 stabilization inhibited intracellular calcium overload and thus prevented CaMKII-mediated cofilin phosphorylation, resulting in decreased EC apoptosis. These data strongly suggest that DAPA-mediated protection of the cardiac microcirculation is due to inhibition of the XO-SERCA2-CaMKII-cofilin pathway.

## Figures and Tables

**Table 1:** The echocardiography parameters in mice treated with DAPA after cardiac ischemia/reperfusion injury

Parameter	Sham	IRI	IRI+DAPA
HR, bmp	463.4 ± 11.2	473.6 ± 13.5	471.8 ± 15.6
LVDd, mm	3.26 ± 0.12	3.98 ± 0.15^#^	3.49 ± 0.11*
LVDs, mm	2.23 ± 0.08	3.12 ± 0.17^#^	2.51 ± 0.12*
IVS, mm	0.84 ± 0.02	0.68 ± 0.04^#^	0.76 ± 0.03*
PW, mm	0.78 ± 0.04	0.79 ± 0.05	0.77 ± 0.03
FS, %	33.8 ± 1.6	20.4 ± 3.1^#^	29.7 ± 1.9*
EF, %	62.4 ± 3.1	43.9 ± 4.6^#^	57.5 ± 4.3*

LVDd, diastolic dimension of left ventricle; LVDs, systolic dimension of left ventricle; IVS, thickness of interventricular septum; PW, thickness of posterior wall; PW, thickness of posterior wall; FS, ratio of left ventricular fractional shortening; HR, heart rate; bmp, beats/min; #p < 0.05 vs. sham, *p < 0.05 vs. IRI.

## References

[1] Davidson SM, Ferdinandy P, Andreadou I, Bøtker HE, Heusch G, Ibáñez B (2019). Multitarget Strategies to Reduce Myocardial Ischemia/Reperfusion Injury: JACC Review Topic of the Week. J Am Coll Cardiol.

[2] Turer AT, Hill JA (2010). Pathogenesis of myocardial ischemia-reperfusion injury and rationale for therapy. Am J Cardiol.

[3] Schulz R, Kelm M, Heusch G (2004). Nitric oxide in myocardial ischemia/reperfusion injury. Cardiovasc Res.

[4] Forman MB, Puett DW, Virmani R (1989). Endothelial and myocardial injury during ischemia and reperfusion: pathogenesis and therapeutic implications. J Am Coll Cardiol.

[5] Kang S, Yang Y (2007). Coronary microvascular reperfusion injury and no-reflow in acute myocardial infarction. Clin Invest Med.

[6] Scheen AJ (2020). Sodium-glucose cotransporter type 2 inhibitors for the treatment of type 2 diabetes mellitus. Nat Rev Endocrinol.

[7] Tahrani AA, Barnett AH, Bailey CJ (2013). SGLT inhibitors in management of diabetes. Lancet Diabetes Endocrinol.

[8] Wiviott SD, Raz I, Bonaca MP, Mosenzon O, Kato ET, Cahn A (2019). Dapagliflozin and Cardiovascular Outcomes in Type 2 Diabetes. N Engl J Med.

[9] McMurray JJV, Solomon SD, Inzucchi SE, Køber L, Kosiborod MN, Martinez FA (2019). Dapagliflozin in Patients with Heart Failure and Reduced Ejection Fraction. N Engl J Med.

[10] Sposito AC, Breder I, Soares AAS, Kimura-Medorima ST, Munhoz DB, Cintra RMR (2021). Dapagliflozin effect on endothelial dysfunction in diabetic patients with atherosclerotic disease: a randomized active-controlled trial. Cardiovasc Diabetol.

[11] Uthman L, Homayr A, Juni RP, Spin EL, Kerindongo R, Boomsma M (2019). Empagliflozin and Dapagliflozin Reduce ROS Generation and Restore NO Bioavailability in Tumor Necrosis Factor α-Stimulated Human Coronary Arterial Endothelial Cells. Cell Physiol Biochem.

[12] Tian J, Zhang M, Suo M, Liu D, Wang X, Liu M (2021). Dapagliflozin alleviates cardiac fibrosis through suppressing EndMT and fibroblast activation via AMPKα/TGF-β/Smad signalling in type 2 diabetic rats. J Cell Mol Med.

[13] Zhang Y, Hu SY, Yin T, Tian F, Wang S, Zhang Y (2015). [Liraglutide promotes proliferation and migration of cardiac microvascular endothelial cells through PI3K/Akt and MAPK/ERK signaling pathways]. Nan Fang Yi Ke Da Xue Xue Bao.

[14] Tan Y, Mui D, Toan S, Zhu P, Li R, Zhou H (2020). SERCA Overexpression Improves Mitochondrial Quality Control and Attenuates Cardiac Microvascular Ischemia-Reperfusion Injury. Mol Ther Nucleic Acids.

[15] Zhou H, Shi C, Hu S, Zhu H, Ren J, Chen Y (2018). BI1 is associated with microvascular protection in cardiac ischemia reperfusion injury via repressing Syk-Nox2-Drp1-mitochondrial fission pathways. Angiogenesis.

[16] Zhou H, Wang J, Zhu P, Hu S, Ren J (2018). Ripk3 regulates cardiac microvascular reperfusion injury: The role of IP3R-dependent calcium overload, XO-mediated oxidative stress and F-action/filopodia-based cellular migration. Cell Signal.

[17] Zhu H, Jin Q, Li Y, Ma Q, Wang J, Li D (2018). Melatonin protected cardiac microvascular endothelial cells against oxidative stress injury via suppression of IP3R-[Ca(2+)]c/VDAC-[Ca(2+)]m axis by activation of MAPK/ERK signaling pathway. Cell Stress Chaperones.

[18] Ni Y, Deng J, Bai H, Liu C, Liu X, Wang X (2022). CaMKII inhibitor KN-93 impaired angiogenesis and aggravated cardiac remodelling and heart failure via inhibiting NOX2/mtROS/p-VEGFR2 and STAT3 pathways. J Cell Mol Med.

[19] Reventun P, Sanchez-Esteban S, Cook A, Cuadrado I, Roza C, Moreno-Gomez-Toledano R (2020). Bisphenol A induces coronary endothelial cell necroptosis by activating RIP3/CamKII dependent pathway. Sci Rep.

[20] Sato D, Uchinoumi H, Bers DM (2021). Increasing SERCA function promotes initiation of calcium sparks and breakup of calcium waves. J Physiol.

[21] Primeau JO, Armanious GP, Fisher ME, Young HS (2018). The SarcoEndoplasmic Reticulum Calcium ATPase. Subcell Biochem.

[22] Qin F, Siwik DA, Lancel S, Zhang J, Kuster GM, Luptak I (2013). Hydrogen peroxide-mediated SERCA cysteine 674 oxidation contributes to impaired cardiac myocyte relaxation in senescent mouse heart. J Am Heart Assoc.

[23] Tong X, Evangelista A, Cohen RA (2010). Targeting the redox regulation of SERCA in vascular physiology and disease. Curr Opin Pharmacol.

[24] Goodman JB, Qin F, Morgan RJ, Chambers JM, Croteau D, Siwik DA (2020). Redox-Resistant SERCA [Sarco(endo)plasmic Reticulum Calcium ATPase] Attenuates Oxidant-Stimulated Mitochondrial Calcium and Apoptosis in Cardiac Myocytes and Pressure Overload-Induced Myocardial Failure in Mice. Circulation.

[25] Toya T, Ito K, Kagami K, Osaki A, Sato A, Kimura T (2020). Impact of oxidative posttranslational modifications of SERCA2 on heart failure exacerbation in young patients with non-ischemic cardiomyopathy: A pilot study. Int J Cardiol Heart Vasc.

[26] Zhao Y, Vanhoutte PM, Leung SW (2015). Vascular nitric oxide: Beyond eNOS. J Pharmacol Sci.

[27] Mei Y, Thompson MD, Shiraishi Y, Cohen RA, Tong X (2014). Sarcoplasmic/endoplasmic reticulum Ca2+ ATPase C674 promotes ischemia- and hypoxia-induced angiogenesis via coordinated endothelial cell and macrophage function. J Mol Cell Cardiol.

[28] Li C, Ma Q, Toan S, Wang J, Zhou H, Liang J (2020). SERCA overexpression reduces reperfusion-mediated cardiac microvascular damage through inhibition of the calcium/MCU/mPTP/necroptosis signaling pathways. Redox Biol.

[29] Li X, Römer G, Kerindongo RP, Hermanides J, Albrecht M, Hollmann MW (2021). Sodium Glucose Co-Transporter 2 Inhibitors Ameliorate Endothelium Barrier Dysfunction Induced by Cyclic Stretch through Inhibition of Reactive Oxygen Species. Int J Mol Sci.

[30] Hussein AM, Eid EA, Taha M, Elshazli RM, Bedir RF, Lashin LS (2020). Comparative Study of the Effects of GLP1 Analog and SGLT2 Inhibitor against Diabetic Cardiomyopathy in Type 2 Diabetic Rats: Possible Underlying Mechanisms. Biomedicines.

[31] Kingir ZB, Özdemir Kural ZN, Cam ME, Cilingir OT, Şekerler T, Ercan F (2019). Effects of dapagliflozin in experimental sepsis model in rats. Ulus Travma Acil Cerrahi Derg.

[32] Arow M, Waldman M, Yadin D, Nudelman V, Shainberg A, Abraham NG (2020). Sodium-glucose cotransporter 2 inhibitor Dapagliflozin attenuates diabetic cardiomyopathy. Cardiovasc Diabetol.

[33] Andersson KB, Finsen AV, Sjåland C, Winer LH, Sjaastad I, Odegaard A (2009). Mice carrying a conditional Serca2(flox) allele for the generation of Ca(2+) handling-deficient mouse models. Cell Calcium.

[34] Abe H, Tanada Y, Omiya S, Podaru MN, Murakawa T, Ito J (2021). NF-κB activation in cardiac fibroblasts results in the recruitment of inflammatory Ly6C(hi) monocytes in pressure-overloaded hearts. Sci Signal.

[35] Zhou H, Wang J, Zhu P, Zhu H, Toan S, Hu S (2018). NR4A1 aggravates the cardiac microvascular ischemia reperfusion injury through suppressing FUNDC1-mediated mitophagy and promoting Mff-required mitochondrial fission by CK2alpha. Basic Res Cardiol.

[36] Coleman RC, Eguchi A, Lieu M, Roy R, Barr EW, Ibetti J (2021). A peptide of the N terminus of GRK5 attenuates pressure-overload hypertrophy and heart failure. Sci Signal.

[37] Wang J, Zhu P, Li R, Ren J, Zhang Y, Zhou H (2020). Bax inhibitor 1 preserves mitochondrial homeostasis in acute kidney injury through promoting mitochondrial retention of PHB2. Theranostics.

[38] Gaspers LD, Thomas AP (2008). Calcium-dependent activation of mitochondrial metabolism in mammalian cells. Methods (San Diego, Calif).

[39] Owen PL, Johns T (1999). Xanthine oxidase inhibitory activity of northeastern North American plant remedies used for gout. J Ethnopharmacol.

[40] Mohamed Isa SSP, Ablat A, Mohamad J (2018). The Antioxidant and Xanthine Oxidase Inhibitory Activity of Plumeria rubra Flowers. Molecules.

[41] Zhang Y, Zhou H, Wu W, Shi C, Hu S, Yin T (2016). Liraglutide protects cardiac microvascular endothelial cells against hypoxia/reoxygenation injury through the suppression of the SR-Ca(2+)-XO-ROS axis via activation of the GLP-1R/PI3K/Akt/survivin pathways. Free radical biology & medicine.

[42] Tanajak P, Sa-Nguanmoo P, Sivasinprasasn S, Thummasorn S, Siri-Angkul N, Chattipakorn SC (2018). Cardioprotection of dapagliflozin and vildagliptin in rats with cardiac ischemia-reperfusion injury. J Endocrinol.

[43] Mizuno M, Kuno A, Yano T, Miki T, Oshima H, Sato T (2018). Empagliflozin normalizes the size and number of mitochondria and prevents reduction in mitochondrial size after myocardial infarction in diabetic hearts. Physiol Rep.

[44] Zhou H, Wang S, Zhu P, Hu S, Chen Y, Ren J (2018). Empagliflozin rescues diabetic myocardial microvascular injury via AMPK-mediated inhibition of mitochondrial fission. Redox Biol.

[45] Gourlay CW, Ayscough KR (2005). The actin cytoskeleton: a key regulator of apoptosis and ageing?. Nat Rev Mol Cell Biol.

[46] McGough A, Pope B, Chiu W, Weeds A (1997). Cofilin changes the twist of F-actin: implications for actin filament dynamics and cellular function. J Cell Biol.

[47] Shi C, Cai Y, Li Y, Li Y, Hu N, Ma S (2018). Yap promotes hepatocellular carcinoma metastasis and mobilization via governing cofilin/F-actin/lamellipodium axis by regulation of JNK/Bnip3/SERCA/CaMKII pathways. Redox Biol.

[48] Arber S, Barbayannis FA, Hanser H, Schneider C, Stanyon CA, Bernard O (1998). Regulation of actin dynamics through phosphorylation of cofilin by LIM-kinase. Nature.

[49] Zhao JW, Gao ZL, Ji QY, Wang H, Zhang HY, Yang YD (2012). Regulation of cofilin activity by CaMKII and calcineurin. Am J Med Sci.

[50] Bhattacharyya M, Karandur D, Kuriyan J (2020). Structural Insights into the Regulation of Ca(2+)/Calmodulin-Dependent Protein Kinase II (CaMKII). Cold Spring Harb Perspect Biol.

[51] Smith GL, Eisner DA (2019). Calcium Buffering in the Heart in Health and Disease. Circulation.

[52] Uthman L, Kuschma M, Römer G, Boomsma M, Kessler J, Hermanides J (2021). Novel Anti-inflammatory Effects of Canagliflozin Involving Hexokinase II in Lipopolysaccharide-Stimulated Human Coronary Artery Endothelial Cells. Cardiovasc Drugs Ther.

[53] Nugrahaningrum DA, Marcelina O, Liu C, Wu S, Kasim V (2020). Dapagliflozin Promotes Neovascularization by Improving Paracrine Function of Skeletal Muscle Cells in Diabetic Hindlimb Ischemia Mice Through PHD2/HIF-1α Axis. Front Pharmacol.

[54] Madonna R, Barachini S, Moscato S, Ippolito C, Mattii L, Lenzi C (2022). Sodium-glucose cotransporter type 2 inhibitors prevent ponatinib-induced endothelial senescence and disfunction: A potential rescue strategy. Vascul Pharmacol.

[55] Sugiyama S, Jinnouchi H, Kurinami N, Hieshima K, Yoshida A, Jinnouchi K (2018). The SGLT2 Inhibitor Dapagliflozin Significantly Improves the Peripheral Microvascular Endothelial Function in Patients with Uncontrolled Type 2 Diabetes Mellitus. Intern Med.

[56] Alshnbari AS, Millar SA, O'Sullivan SE, Idris I (2020). Effect of Sodium-Glucose Cotransporter-2 Inhibitors on Endothelial Function: A Systematic Review of Preclinical Studies. Diabetes Ther.

[57] Zainordin NA, Hatta S, Mohamed Shah FZ, Rahman TA, Ismail N, Ismail Z (2020). Effects of Dapagliflozin on Endothelial Dysfunction in Type 2 Diabetes With Established Ischemic Heart Disease (EDIFIED). J Endocr Soc.

[58] Pedersen MG, Ahlstedt I, El Hachmane MF, Göpel SO (2016). Dapagliflozin stimulates glucagon secretion at high glucose: experiments and mathematical simulations of human A-cells. Sci Rep.

[59] Adingupu DD, Göpel SO, Grönros J, Behrendt M, Sotak M, Miliotis T (2019). SGLT2 inhibition with empagliflozin improves coronary microvascular function and cardiac contractility in prediabetic ob/ob(-/-) mice. Cardiovasc Diabetol.

[60] Cook NL, Viola HM, Sharov VS, Hool LC, Schöneich C, Davies MJ (2012). Myeloperoxidase-derived oxidants inhibit sarco/endoplasmic reticulum Ca2+-ATPase activity and perturb Ca2+ homeostasis in human coronary artery endothelial cells. Free radical biology & medicine.

[61] Evangelista AM, Thompson MD, Bolotina VM, Tong X, Cohen RA (2012). Nox4- and Nox2-dependent oxidant production is required for VEGF-induced SERCA cysteine-674 S-glutathiolation and endothelial cell migration. Free radical biology & medicine.

[62] Willer EA, Malli R, Bondarenko AI, Zahler S, Vollmar AM, Graier WF (2012). The vascular barrier-protecting hawthorn extract WS® 1442 raises endothelial calcium levels by inhibition of SERCA and activation of the IP3 pathway. J Mol Cell Cardiol.

[63] Pernomian L, do Prado AF, Silva BR, de Paula TD, Grando MD, Bendhack LM (2021). C-type natriuretic peptide-induced relaxation through cGMP-dependent protein kinase and SERCA activation is impaired in two kidney-one clip rat aorta. Life Sci.

[64] Qin F, Siwik DA, Pimentel DR, Morgan RJ, Biolo A, Tu VH (2014). Cytosolic H2O2 mediates hypertrophy, apoptosis, and decreased SERCA activity in mice with chronic hemodynamic overload. Am J Physiol Heart Circ Physiol.

[65] Zhou H, Hu S, Jin Q, Shi C, Zhang Y, Zhu P (2017). Mff-Dependent Mitochondrial Fission Contributes to the Pathogenesis of Cardiac Microvasculature Ischemia/Reperfusion Injury via Induction of mROS-Mediated Cardiolipin Oxidation and HK2/VDAC1 Disassociation-Involved mPTP Opening. J Am Heart Assoc.

[66] Belosludtsev KN, Starinets VS, Belosludtsev MN, Mikheeva IB, Dubinin MV, Belosludtseva NV (2021). Chronic treatment with dapagliflozin protects against mitochondrial dysfunction in the liver of C57BL/6NCrl mice with high-fat diet/streptozotocin-induced diabetes mellitus. Mitochondrion.

[67] He L, Li Y, Zhang D, Song H, Xu D, Song Z (2022). Dapagliflozin improves endothelial cell dysfunction by regulating mitochondrial production via the SIRT1/PGC-1α pathway in obese mice. Biochem Biophys Res Commun.

[68] Lahnwong S, Palee S, Apaijai N, Sriwichaiin S, Kerdphoo S, Jaiwongkam T (2020). Acute dapagliflozin administration exerts cardioprotective effects in rats with cardiac ischemia/reperfusion injury. Cardiovasc Diabetol.

